# Early-onset catatonia associated with *SHANK3* mutations: looking at the autism spectrum through the prism of psychomotor phenomena

**DOI:** 10.3389/fpsyt.2023.1186555

**Published:** 2023-09-22

**Authors:** Dirk Dhossche, Clément de Billy, Claudine Laurent-Levinson, Marie T. Le Normand, Christophe Recasens, Laurence Robel, Anne Philippe

**Affiliations:** ^1^Department of Adolescent Psychiatry, Inland Northwest Behavioral Health, Spokane, WA, United States; ^2^CEMNIS – Noninvasive Neuromodulation Center, University Hospital Strasbourg, Strasbourg, France; ^3^Faculté de Médecine Sorbonne Université, Groupe de Recherche Clinique no. 15 – Troubles Psychiatriques et Développement (PSYDEV), Paris, France; ^4^Centre de Référence des Maladies Rares à Expression Psychiatrique, Département de Psychiatrie de l’enfant et l’adolescent, Hôpital Pitié-Salpétrière, Paris, France; ^5^Institut de l’Audition, Institut Pasteur, Paris, France; ^6^Laboratoire de Psychopathologie et Processus de Santé, Université de Paris Cité, Paris, France; ^7^Service universitaire de Psychiatrie de l’Enfant et de l’Adolescent, Centre hospitalier Intercommunal de Créteil, Créteil, France; ^8^Unité de Psychopathologie de l’Enfant et de l’Adolescent, GHU Paris, Psychiatrie et Neurosciences, Hôpital Sainte Anne, Paris, France; ^9^Université Paris Cité, Paris, France; ^10^INSERM U1163 Institut Imagine, Paris, France

**Keywords:** *SHANK3*, Phelan McDermid syndrome, 22q13.3 deletion, catatonia, autism spectrum disorder, Wernicke-Kleist-Leonhard school, Heller, abnormal movement

## Abstract

**Background:**

Individuals with Phelan-McDermid syndrome (PMS) present with a wide range of diagnoses: autism spectrum disorder, intellectual disability, or schizophrenia. Differences in the genetic background could explain these different neurodevelopmental trajectories. However, a more parsimonious hypothesis is to consider that they may be the same phenotypic entity. Catatonic disturbances occasionally reported from adolescence onwards in PMS prompts exploration of the hypothesis that this clinical entity may be an early-onset form of catatonia. The largest cohort of children with childhood catatonia was studied by the Wernicke-Kleist-Leonhard school (WKL school), which regards catatonia as a collection of qualitative abnormalities of psychomotricity that predominantly affecting involuntary motricity (reactive and expressive). The aim of this study was to investigate the presence of psychomotor signs in three young adults carrying a mutation or intragenic deletion of the *SHANK3* gene through the prism of the WKL school conception of catatonia.

**Methods:**

This study was designed as an exploratory case study. Current and childhood psychomotor phenomena were investigated through semi-structured interviews with the parents, direct interaction with the participants, and the study of documents reporting observations of the participants at school or by other healthcare professionals.

**Results:**

The findings show catatonic manifestations from childhood that evolved into a chronic form, with possible phases of sub-acute exacerbations starting from adolescence.

**Conclusion:**

The presence of catatonic symptoms from childhood associated with autistic traits leads us to consider that this singular entity fundamentally related to *SHANK3* mutations could be a form of early-onset catatonia. Further case studies are needed to confirm our observations.

*“Pluralitas non est ponenda sine necessitate.”*

*Guillaume d’Occam (1285-1347)*

## Introduction

The *SHANK3* gene is located at the end of the long arm of chromosome 22. Ring chromosomes 22 and 22q13.3 deletions (1), as well as *SHANK3* pathogenic sequence variants (2, 3), lead to disruption of the SHANK3 protein, which is essential for organizing the post-synaptic density of glutamatergic excitatory synapses, expressed in the central nervous system as well as in the neuromuscular junction and the dendrites of sympathetic postganglionic neurons and myenteric neurons (4).

In 2001, Phelan et al. (5) reported neonatal hypotonia, global developmental delay, and strongly impaired speech and expressive language, as well as autistic traits, in the absence of major dysmorphisms in patients carrying 22q13.3 deletions. This clinical picture has since been known as Phelan-McDermid syndrome (PMS). Indeed, a number of early negative signs, specifically, the absence of skills related to typical development, in particular, communication skills, such as not responding to one’s name or language delay, may suggest an autism spectrum disorder in children with PMS (6).

However, careful study of the clinical manifestations of these children has shown a disparity between the autistic traits of PMS and the prototypical autism described by Kanner (7). First, positive signs are absent, i.e., signs that do not appear during typical development, such as the deviant development of language, with *delayed* echolalia, pronominal inversion, idiosyncratic language, atypical visual exploratory behaviour, and idiosyncratic concerns, etc. (8). Second, the presence of atypical signs or an unusual course differs from Kanner’s clinical description. In particular, PMS individuals feature psychomotor behavioural abnormalities, such as facial expressions that are fixed for several minutes (catatonia-like) (9), episodes of “hypotonia” without the alteration of consciousness for several hours, and rapid bursts of blinking for several minutes (7), etc. Furthermore, the loss of functional daily living, communication, socialisation or motor skills can occur during infancy, adolescence, or adulthood for PMS individuals (10–18).

Differences between PMS and prototypical autism have recently been supported by functional neuroimaging and neurophysiological studies (19). Ponson et al. (20) did not find shortening of the latency of the MMN (*mismatch negativity*) wave in 10 individuals with PMS, including those who met ADI-R (Autism Diagnostic Interview-Revised) and ADOS-2 (Autism Diagnostic Observation Schedule, Second Edition) threshold scores for clinical criteria for autism spectrum disorder (ASD). Such shortening is generally observed in cases of prototypical autism (21).

Direct sequencing of the *SHANK3* gene has shown mutations in 0.5% of individuals with ASD (2, 3), 0.5 to 1% of individuals with intellectual disability (22–24), and 0.5% of individuals with schizophrenia (25, 26), supporting the significant clinical heterogeneity according to DSM-5. Moreover, we previously reported a case of Heller disintegrative disorder in a girl with an intragenic deletion of the *SHANK3* gene (12).

Such nosographic multiplicity may correspond to different mutations in the *SHANK3* gene (27, 28), additional genetic or epigenetic factors (29–32), or to the presence of non-genetic factors (exposure to toxic substances *in utero*, neonatal distress, psychosocial risk factors) (33) with a different pathophysiological impact, thus accounting for these different neurodevelopmental trajectories (34). However, the question arises as to how this can be possible for 18 individuals carrying the same recurrent frameshift p.Ala1227Glyfs*69 variant in *SHANK3* and whose whole genome sequence does not reveal other candidate variants, either rare or frequent (35).

A more parsimonious hypothesis is to consider that it may be the same rare phenotypic entity. The common origin of these disorders forces us to search for a conceptual framework to provide semiological coherence with the psychobehavioural disturbances of these individuals. The “reverse phenotyping” approach leads to careful re-examination of the phenotype of these individuals who share the same genotype by focusing on finding a frame of reference that favours the detection of discriminating signs or symptoms from the viewpoint of a comparative clinical analysis rather than describing general symptoms (36, 37). Current international classifications based on an epidemiological semiology generate different diagnoses but do not take into account unusual signs that support common links and continuities between seemingly different diagnoses (38).

Catatonic manifestations, often reported from adolescence onwards, especially for individuals carrying pathogenic sequence variants or intragenic deletions of the *SHANK3* gene (14–17), prompt us to explore the hypothesis that this rare clinical entity could be a form of early catatonia by virtue of the principle of Occam’s razor, applied here to the entirety of an individual’s life, and to re-examine the history of the various conceptions of this syndrome since its first clinical description.

Catatonia is a mysterious phenomenon, which may explain its chequered history in adults and children (39–41). It was first clinically conceptualised as a disease of the brain in 1874 by Karl Ludwig Kahlbaum (1828–1899), who described it as an autonomous pathological entity that can occur in childhood. The individualisation of catatonia is based, alongside a singular psychological symptomatology, on *muscular signs*, “which are of essential importance in the general configuration of the morbid process” and on *an alternating and cyclical course*, consisting of different phases likely to be repeated according to a more-or-less regular pattern, with successive phases of melancholy, mania, and stupor, which constitute the unitary element, before ending in recovery or “confusion-stupidity” (40, 42–45).

It was then included in 1899 as a disorder of the will under the influence of Emil Kraepelin (1856–1926) in the sphere of dementia praecox, later called schizophrenia, and Eugen Bleuler (1857–1939), who reinterpreted the signs of catatonia with a psychological orientation.

Finally, from the 1970s on, the publication of a series of cases of catatonia found in affective or organic psychoses led to its repositioning as a transnosographic syndrome, either as a separate “idiopathic” syndrome or as a specifier of the course of other psychiatric disorders (psychotic disorder, developmental disorders, including ASD) or general medical conditions (46, 47).

In children, cases of catatonia were published in the first decades of the twentieth century, mainly in the German scientific literature (41). Analogous to the history of this concept in adults, catatonia in children became dominated by multiple diagnoses, such as hebephrenia (48), dementia infantilis (49, 50), pervasive refusal syndrome (51, 52), conversion disorder (53, 54), and autism (55, 56).

The largest cohort of catatonic children was studied by Karl Leonhard (1904–1988), who together with Carl Wernicke (1848–1905), and Karl Kleist (1879–1960) constituted the Wernicke-Kleist-Leonhard school (WKL school). Leonhard defined early childhood catatonia based on more than 100 cases of catatonia that had begun in the first years of life among children considered to be autistic or intellectually disabled (57).

Our aim was to investigate the presence of psychomotor signs in three young adults carrying a mutation of the *SHANK3* gene or an interstitial 22q13 deletion encompassing exons 10 to 22 of the *SHANK3* gene, with a particular focus on the psychomotor phenomena described by the WKL school. The term “psychomotor” refers to several conceptions (40). For the WKL school, it applies to involuntary motor actions that have the character of intentional acts but are carried out without the influence of will, namely expressive psychomotricity (facial mimics, body postures, etc.) and reactive psychomotricity, which corresponds to mimics, postures and motor acts of speech in response to external stimuli as well as learned social behaviours (e.g., directing one’s gaze towards one’s interlocutor or grasping the hand of one’s interlocutor in adulthood in order to greet him).

## Materials and methods

The present study was designed as an exploratory case study. The aim was to explore psychomotor phenomena. We present a brief description of the system catatonias described by the WKL school due to their limited diffusion in the Anglo-Saxon literature.

The WKL school relied on meticulous clinical studies of a very large number of patients followed for years, often for their entire lives, before the arrival of the first psychotropic drugs, allowing the observation of their natural history to develop a classification of “endogenous” psychoses that includes 35 nosographic entities divided into five categories: unipolar and bipolar episodic affective psychoses, cycloid psychoses, system schizophrenias, and non-system schizophrenias. These entities show good reliability, with good inter-rater reproducibility, and good stability, with good test–retest reproducibility (58). Genetic and functional brain imaging studies are being undertaken to establish their validity (59, 60).

This classification is based on the recognition of distinct symptom-complexes, i.e., on a precise combination of certain specific symptoms that characterize these phenotypes from a semiology comprising over 200 signs and symptoms. Unlike the DSM approach which diagnoses a disorder, the WKL diagnosis is a stable lifelong diagnosis, so it must be able to explain the current clinical presentation, take into account the history of the illness and remain consistent with subsequent episodes (58). It is less known, less used, and more complex than the DSM.

### Overview of system catatonia in the WKL classification

The phenomenology of psychomotor anomalies studied successively by Wernicke, Kleist and Leonhard (WKL school) led Leonhard to describe six simple forms of system catatonia in adults and then, to identify the corresponding clinical pictures in children whose motricity, emotional lability and language development significantly affect the clinical presentations relative to those of adults (37, 57, 61).

These six simple forms of system catatonia are organised into three pairs with globally opposite symptom complexes: parakinetic (excessive spontaneous movements) versus pseudo-compulsive (deficient spontaneous movements), negativistic (opposition to environmental stimuli) versus proskinetic (adherence to the environment), and speech-prompt (short-circuiting responses) versus sluggish (slowness of responses) (37, 57).

The diagnosis of such system catatonia is based on the recognition of specific psychomotor signs, such as proskinesia, gegenhalten, parakinesia, prompt-response, and pseudo-compulsion, coupled with other less specific but relatively stable psychiatric symptoms forming unique gestalts of psychiatric syndromes (40).

The WKL model assumes the existence of psychomotor brain centers linking the intrapsychic systems (i.e., affects, will, and thoughts) and the motor systems. These psychomotor centers receive various intrapsychic inputs from conscious and non-conscious processes and convert them into coherent behavioural outputs that are transmitted to the motor systems. The motor systems are responsible for the translation and fluid coordination of these movements, actions, and behaviours of orientation, which are reactive and expressive, including motor acts of speech (40). The observation of a possible double automatic-voluntary dissociation between voluntary facial motor skills and involuntary expressive facial motor skills (automatic-voluntary dissociation in a lesion of the primary cortex, with damage to voluntary facial motor skills alone while expressive facial motor skills are spared, and rare cases of inverse automatic-voluntary dissociation, with damage to expressive facial motor skills while voluntary facial motor skills are preserved) suggests the existence of psychomotor centers (62, 63).

The WKL-school emphasises the distinction between *qualitative* and *quantitative* psychomotor abnormalities. Qualitative abnormalities (negativism with ambitendency, gegenhalten,^1^ mitgehen,^2^ stereotypies, posture maintenance, parakinesia, proskinesia, pseudo-compulsions etc.) indicate a dysfunction of the psychomotor centers, whereas quantitative abnormalities, i.e., a decrease (akinesia) or an increase (hyperkinesia) in reactive or expressive motricity but without distortion of movement, are due to hypo-or hyperfunctioning of the psychomotor systems, explained by an alteration of the regulatory systems rather than by a dysfunction of the psychomotor centers themselves. According to the WKL school, system catatonia is due to a dysfunction of the psychomotor centers and therefore results in qualitative abnormalities of psychomotricity.

*Parakinetic catatonia* is characterised by parakinesias, i.e., extremely variable involuntary movements that are not spontaneously perceived by the subject, and which distort his psychomotricity, giving the impression of strangeness (64, 65). Such parakinesia can take the form of “pseudo-expressive” movements (a fleeting mimicry characterised by a “smiling” facial expression that is not congruent with the emotional state), “pseudo-reactive” movements (a shrug of the shoulders or brief body swaying induced by the excitement of an interview) or “pseudo-dystonic” movements (“snout spasms”). This particular disturbance of motor sequences is also reflected in speech by short nongrammatical responses and words emitted in isolated bursts. It contrasts with *pseudo-compulsive catatonia*, in which pseudo-compulsion predominates at the beginning of the evolution. Such pseudo-compulsion consists of rigid and essentially non-modifiable complex gestural sequences without apparent reason. They are not underpinned by congruent obsessive ideas and are not criticised by the individual, and have the appearance of rituals, stereotypies or compulsions. The subject may be irritated if prevented from performing them but there is no anxiety involved. The egodystonic character is therefore less apparent and the level of insight is generally reduced, which differentiates them from obsessive–compulsive disorders. It evolves towards increasing impoverishment of involuntary, reactive, and expressive motor skills, leading to a rigid posture and rigid movements.

*Negativistic catatonia* results in automatic behavioural resistance in response to external stimulation (psychomotor negativism), whereas *proskinetic catatonia* reveals an abnormal tendency to automatically initiate movements in response to external stimulation. It can be manifested by (i) the fact that individuals turn towards their interlocutor with a facial expression that, despite being an empty mimicry, shows some kind of interest, and begin mumbling incomprehensively (verbigeration), (ii) automatic turning towards objects and meaningless manipulation (utilisation behaviour), and (iii) exaggerated turning towards events in the environment or (iv) “responsive grasping “from adulthood onwards, corresponding to an inability of the individual to inhibit the hand shaking gesture when the hand is held out, despite the indication not to shake it (“impulse-automatisms”).

*Sluggish catatonia* is defined by an orientation of the subject’s attention towards his or her internal psychic life (his or her hallucinations), resulting in low reactivity to the environment (slowness of responses), and an absent air (apathy), in contrast to *speech-prompt catatonia*, in which the lack of enthusiasm to speak gives way as the subject is stimulated (warm up effect) to short circuit answers that are sometimes meaningless. The subject often gives correct answers to simple questions for which the answer is automatic because it does not require any reflexive activity. On the other hand, more difficult questions lead to side responses, reflecting “short-circuit” responses, i.e., responses that do not result from reflective activity but are produced by the verbalisation of the material that happens to be most ready to be vocalised (66).

In addition to these six simple forms of catatonia, Leonhard also described combined forms, i.e., forms in which two symptomatic complexes of simple catatonia are expressed simultaneously and influence each other (for example catatonia with speech-prompt-parakinetic catatonia, or proskinetic-negativistic catatonia) (37, 57).

Leonhard specified the clinical picture of children relative to adults by describing the particular phenomenology of contact in catatonic children by a subjective feeling in the manner of Rümke’s ‘praecox feeling’ due to the absence of natural expressive movements: “The patients look with an empty expressionless face from which one cannot tell what their inner attitude is in connection with their spoken utterances,” “One never can find out what is going on inside a child with catatonia. They always seem foreign to us. One may infer nothing from their facial expressions. their faces are like masks.” At the same time, the child’s emotional lability can lead to the possibility of expression of emotion in an exaggerated manner (“even vivid laughter appears mask-like”), whereas the basic facial expression is most often hypo-expressive (57).

Moreover, due to the delay in language development, proskinesia, which usually manifests itself in adults as mumbled verbalisations, presents itself in children without language as restlessness, especially noticeable with odd fiddling and grasping movements without an acknowledged purpose (“groped for everything she saw, handled it briefly, then turned towards the next object”), referred to as “automatic rotation towards objects” (57).

Finally, rare combined forms with three symptomatic complexes of simple catatonia appear to be seen only in early childhood catatonia (57).

### Participants

This exploratory study concerned three young adults A, B, and C, aged 23, 25, and 30 years, respectively, carrying of a mutation or intragenic deletion of the *SHANK3* gene, and followed in the genetics department of the Necker-Enfants Malades Hospital from the ages of 3, 5, and 22, respectively. Participants B and C had been followed by one of the authors (AP).

As these psychomotor phenomena are still poorly identified in childhood and, moreover, often subtle and fluctuating, the participants were chosen according to the possibility of establishing an authentic reciprocal relationship with their parents, inspired by the approach of the “consultant” parent becoming a “co-researcher” parent in an attempt to describe with them certain unusual behaviours of their child (67).

The genomic anomalies, perinatal history, development of psychomotor, language and cognitive skills, level of education, autism symptomatology, and brain MRI of the participants are described in Table 1. These data were collected retrospectively from the parents, which explains the discrepancy with the child’s follow-up. Case B has been reported in four case series (1 (patient 18), 7 (patient 1), 68, 69 (case 2)) and case A was reported in one (69 (case 1)). The parents provided written informed consent for the publication of these cases and for the videos.

**Table 1:** Summary of the main characteristics of the three participants.

Participant	A	B	C
Gender, age	M, 23 years	F, 25 years	F, 30 years
Genetic abnormality	Chr22(GRCh37/hg19): g.51160025_51160037del c.3812_3824del, p.Arg1271Leufs*25, deletion of 12 b in exon 22,	Chr22(GRCh37/hg19): 51,122,392–51,178,324 × 1 Interstitial deletion of 55,9 kb encompassing exons 10 to 22 of the SHANK3 gene and exons 1 to 3 of the ACR gene	Chr22(GRCh37/hg19): 51,122,452–51,193,650 × 1 Interstitial deletion of 71,2 kb encompassing exons 10 to 22 of the SHANK3 gene and the whole ACR gene
	*de novo*	*de novo*	Non-maternal inheritance
	Diagnosed at age 15	Diagnosed at age 5	Diagnosed at age 23
Pregnancy and birth	Normal pregnancy and delivery at 39 gestational weeks	Normal pregnancy and delivery at 39 gestational weeks + 4 days	Pregnancy with premature labour at 7 months treated by salbutamol and delivery at 37 gestational weeks
	Weight: 3170 g, length: 50 cm,	Weight: 3400 g; length: 53 cm;	Weight 2,890 g, length: 48 cm,
	head circumference 35.5 cm	head circumference: 35 cm	head circumference: 32 cm
Psychomotor development	Axial hypotonia, sitting at around 9 months, walking at 15 months, broad-based and unsteady gait until 7 years	Sitting at 9 months, walking at 15 months but soon started running with many falls, seemed insensitive to pain	Sitting at around 6 months, walking at 11 months
	Toilet training	Toilet training	Toilet training
	Daytime: 3.5 y	Daytime: 4 y	Daytime: 2.5 y
	Nighttime: 8.5 y	Nighttime: 14 y	Nighttime: 4 y
Language development	A few rough words at 2 years but quickly disappeared, then makes sounds but no syllables.	First words at 10 months, then stagnation and regression around 18 months to 2 years (fewer sounds, no focus, no response to her name), then reappearance of some words around 3–4 years and first sentences around 7 years	First simple words at 5 years and first sentences around 7 years
	First words at 6 years, first sentences at 7 years		
	Oral-facial apraxia		
Cognitive assessment	**PEP-3 (11 y 8 m):**	**WISC-III (8 y 8 m):**	Not performed
	Cognitive verbal/preverbal: 6 y 1 m	Picture completion: 1	
	Expressive language: 4 y 7 m	Information: 1	
	Receptive language: 4 y 4 m	Similarities: 3	
	Fine motor: 4 y 3 m	Picture arrangement: 2	
	Gross motor: 3 y 2 m	Arithmetic: 1	
	Visual-motor imitation: 3 y 1 m	block design: 3	
	Personal autonomy: 5 y 11 m	Vocabulary: 1	
	**K-ABC (11 y 8 m):**	Object assembly: 11	
	Achievement scale: 4 y 11 m	Verbal IQ: 46	
	Faces and places: 6 y	Performance IQ: 60	
	Arithmetic: 4 y 9 m	Full scale IQ: 51	
	Riddles: 2 y 5 m		
	Reading/decoding: 6 y 3 m		
	**WNV (17 y 1 m):**	**WNV (18 y):**	
	Matrices: 11	Matrices: 40	
	Coding: 10	Coding: 10	
	Spatial Span: 10	Spatial Span: 11	
	Picture arrangement: 11	Picture arrangement: 17	
	Full scale: 30 (percentile rank < 0.1)	Full scale: 41 (percentile rank < 0.1)	
Education	Part-time schooling from age 3 to 17 years due to high susceptibility to tiredness.	Kindergarten with a school life assistant until the age of 7, then primary school in a school specialising in language disorders, then part-time schooling until the age of 16 in localized units for school inclusion (ULIS).	Regular schooling until the age of 10 (repeating the first year of primary school), then schooling for one year in a localized unit for school inclusion (ULIS), then home schooling with correspondence courses with the support of her mother.
	Kindergarten with a school life assistant, then primary and secondary education in localized units for inclusive education (ULIS)		
**ADI-R**			
Social interaction: (cut-off 10)	20	18	18
Communication: (cut-off 8)	18	11	22
Restricted and stereotyped interests: (cut-off 3)	2	2	2
**ADOS-2**	ADOS-2-module 2 (16 y 5 m)	Not performed	ADOS-2-module 1 (23 y 9 m)
	(autism cut-off = 9; ASD cut-off = 8)		(autism cut-off = 16; ASD cut-off = 11)
	Social Affect score = 14		Social Affect score = 16
	Repetitive Restricted Behavior score = 0		Repetitive Restricted Behavior score = 4
	Total score = 14		Total score = 20
**Brain MRI**	Small white matter signal abnormalities adjacent to the two occipital horns of the lateral ventricles (non-specific)	No abnormalities	Not performed
	Right temporal arachnoid cyst		

PEP-3, Psychoeducational Profile, 3rd Edition (Schopler E, Lansing MD, Reichler RJ, Marcus LM. (2005); Psychoeducational Profile: TEACCH Individualized Assessment for Children with Autism Spectrum Disorders – Third Edition. Pro-Ed Inc.); WISC-III, Wechsler Intelligence Scale for Children-Third Edition (Wechsler D. Wechsler Intelligence Scale for Children-Third Edition: Canadian (WISC-III). Psychological Corporation. ed. Toronto, Canada1991); K-ABC, Kaufman Assessment Battery for Children (Kaufman, A.S. and Kaufman, N.L. (1983). Kaufman Assessment Battery for Children. Circle Pines, MN: American Guidance Service); WNV, Wechsler Non-Verbal Scale of Ability (Wechsler D., Naglieri J. A., Petermann F. (2006). Wechsler Nonverbal Scale of Ability. San Antonio, TX: PsychCorp); ADI-R, Autism Diagnostic Interview-Revised (Lord C, Rutter M, Le Couteur A. Autism Diagnostic Interview-Revised: a revised version of a diagnostic interview for caregivers of individuals with possible pervasive developmental disorders. J Autism Dev Disord. 1994; 24(5): 659–85); ADOS-2, Autism Diagnostic Observation Schedule, 2nd Edition (Lord C, Rutter M, DiLavore P, Risi S, Gotham K, & Bishop S, Autism Diagnostic Observation Schedule – 2nd edition (ADOS-2). Western Psychological Corporation. 2012).

### Clinical assessment

Psychomotor phenomena can be observed generally in mimicry, behaviour, the way the participant interacts with his or her surroundings and his or her muscle tone at rest. We examined the participants using a phenomenological approach considering that “patients do not manifest a series of independent symptoms or signs, but rather, their symptoms and signs are interdependent and mutually implicative, forming certain meaningful wholes that are interpenetrated by experiences, feelings, expressions, beliefs, and actions, all permeated by biographical detail” to try to adequately translate the patient’s experience, lived in the first person perspective, into the third-person (70). We thus considered three points of view.

First, we sought information on the psychomotor phenomena, in general, as well as those more specifically described by WKL (e.g., parakinesia, proskinesia), through the intermediary of the parents through semi-structured interviews. The presence of these phenomena was assessed based on examples of such phenomena experienced by the parents by asking them to specify the circumstances, their frequency, or other modalities rather than a binary yes/no answer. When parents used psychiatric vocabulary, e.g., ‘stereotypies’, they were asked to precisely describe what they had observed to be as close as possible to reality. Parents were also encouraged to review their home movies to recall their child’s psychomotor characteristics during childhood. As this was an exploratory study with the aim of describing a new and rare clinical entity, any motor phenomenon, however unusual, insignificant, or rare, was sought and taken into account. These interviews took place in the presence of the participant, allowing, on occasion, joint observations of certain behavioural, affective, or psychomotor characteristics with the parents and the sharing of their perception with that of the clinician. For the phenomena mentioned during the interviews but not manifested during them, parents were encouraged to film them with their smartphone at home to better objectify them.

Second, we sought further insights by a direct exchange with the participant, which consisted of observation of the behaviour (facial expression, orientation of the head, parikinesias), both passively during the exchanges with the parents and actively by interacting with him/her through verbal solicitations or through a game, and a physical examination to search for echopraxia, gegenhalten, utilisation behaviour by placing objects on the desk to see how the subject grasps them, etc. (71–73). Participants 2 and 3, whose verbal exchanges were limited during the hospital consultations, were recorded at home with their and their parents’ agreement using the LENA (Language ENvironment Analysis) system, which allows a quasi-automatic verbatim transcription (74).

Finally, we sought further information by (re-)reading documents reporting observations of the participant by teachers or healthcare professionals to retrospectively search for signs that could be interpreted as psychomotor signs in early childhood. These signs were discussed again with the parents.

The interviews were conducted by AP, a senior consultant clinical and research psychiatrist, who had attended Dr. Jack Foucher’s seminar on differential psychopathology of endogenous WKL psychoses at the Strasbourg Hospital (75).

The results are reported in the form of clinical vignettes and a summary table of psychomotor manifestations and retrospective Pediatric Catatonia Diagnostic Scale (PCRS) scoring at the age of 5 to 6 years (76) (Table 2).

**Table 2:** Qualitative psychomotor abnormalities of individuals A, B, and C according to age.

	A	B	C
<6 years of age	Automatic turning towards objects	Automatic turning towards objects	Lack of initiative
		Immediate echolalia (last sound)	Lack of responsiveness to prompting combined with a fixed smile
		Negativism	Mutism
Between 6 and 13 years of age	Automatic turning towards objects	Immediate echolalia (last sound)	Lack of initiative
	Posturing	Echopraxia	Fixed smile
		Blinking	Latency of responses
		Mutism	Mutism
		Negativism	Automatic reading
		Ambitendency	
		Expressionless face but good eye contact	
		Mannerism	
Between 13 and 18 years of age	Posturing	Posturing	Lack of initiative
	Psychomotor slowdown	Pseudo-compulsions	Latency of responses
		Mutism	Mutism
		Rigidity	Automatic reading
		Stiff face	Impulsivity
>18 years of age	Posturing	Negativism	Lack of initiative
	Prompt-responses	Proskinesia	Proskinesia
	Short-circuiting responses	Pseudo-compulsions	Frozen smile
	Pseudo-compulsions	Impulsivity	Sneer
	Negativism	Mannerism (“hands placed symmetrically like an open book”)	Latency of responses
	Impulsive actions	Expressionless face	Mutism
	Utilization Behaviour	Sneer	Verbigeration
	Proskinesia	Mitgehen	Utilization behaviour
	Parakinesias		Automatic reading
	No gegenhalten,		Rubbing of the palms of the hands
	No flexibility cerea		
PCRS around age 6 (threshold > 9)	3	7	6

PCRS, pediatric catatonia diagnostic scale.

## Results

### Case A

A was a calm and smiley baby. From 9 months of age, the parents were concerned about hypotonia and difficulties in controlling his movements and manipulating objects. Walking was acquired at 15 months with an uncoordinated gait and an increase in the width of the base of support. At 2 years, he could say “babe, aica, ma, ma” with gurgles, but these first gurgles disappeared in the following months.

At 2 years 8 months, A did not appear to understand simple instructions. He showed moderate hyperactivity with significant clumsiness: “he falls a lot, bumps into objects,” his movements were jerky and abrupt. He tended to scatter toys or books systematically, throwing them on the floor. He could seek interactions with other children but they did not understand his overly direct way of relating. He drooled, made rather piercing, undeveloped sounds, and shouted a lot with appropriate laughter. He slept well and ate everything with great gusto, but had difficulty chewing.

At 6 years of age, he babbled to express himself, a few words were recognisable, such as “again,” “daddy,” and “mummy,” and he knew how to make himself understood by gestures. Eye contact was of good quality and joint attention capacities were present. Motor agitation was still strong and he went from one object to another without paying attention to his environment. He manipulated wooden toys and cars and threw them, but without constructing an organised sequence or finding meaning in the object itself. He climbed on furniture without considering the consequences of his gestures. Methylphenidate was tried but was stopped as it worsened the agitation. His gait was peculiar, with forearms bent at 90°, so much so that his mother regularly “unfolded” his arms.

From the age of 8 to 9 years, language had developed considerably but the answers to questions were not necessarily adapted because he had difficulty taking into account what the other person was saying. He often talked about his cartoons. Indeed, he was fascinated by the DVDs, which he tended to repeatedly recall aloud and which he ended up knowing by heart. He enjoyed going to the cinema, the zoo, or museums. During guided tours, when the guide asked the audience if there were any questions, A always asked a very appropriate question, which always surprised his mother. He exhibited bilateral twitches of the upper limbs, with rapid rotation of the wrists and hands in response to positive stimuli, for example, when he was congratulated.

At the age of 16, A presented a sudden break in behaviour with a global psychomotor slowdown, a sad face, anhedonia, apragmatism, and hypersomnia. Treatment with sertraline was prescribed for possible underlying depression and anxiety, but quickly stopped due to poor tolerance, and A’s condition rapidly returned to its previous state.

At the age of 18, A moved into a group home with 5 to 6 other young people, which he did not like, and the adaptation proved to be very difficult. Indeed, as soon as A discovered his room, he immediately settled on his bed and did not move, refusing any interaction whatsoever: refusal to eat, get up, or go to the toilet, etc. The educational team used reinforcers (DVDs) without success. He lost weight and food supplements were given to him when he accepted them.

For more than 2 years, the adaptation was difficult and A’s behaviour showed major fluctuations, alternating between episodes of exacerbated and less marked opposition, which led to an 18 month hospitalisation. A developed ritualised gestural sequences lasting 20 to 30 s in which he appeared unapproachable, quietly moving his upper limbs in a repeated up and down movement with great amplitude, making onomatopoeia, and repeating words in a stereotyped manner (Supplementary Video 1).

Despite various treatments (aripiprazole, lorazepam, lithium, transcranial direct-current stimulation), which resulted in short-lived improvements, attempts to reintegrate him into his group home proved unsuccessful and he finally returned to his home while waiting for a more medicalised adapted structure.

Back at home, he refused to go outside. When he got frustrated and angry, he yelled but he never hit or broke anything. Occasionally he scratched the floor with his nails repeatedly for no apparent reason (Supplementary Video 2). He sometimes had verbal impulses that lasted less than 10 s, without any apparent trigger, in which he came within a few centimetres of his mother’s face, frowning and yelling; she was terrified but managed to ask him by forcing herself to smile “Are you okay A?” and the angry expression on her son’s face disappeared as quickly as it came.

A showed great interest in the interview situation. He spontaneously asked questions (“how old are you?”) to get in touch with his interlocutor. However, his speech was difficult to follow because of syntactic disorders (a sentence could be immediately followed by its negation, “I remember,” “I do not remember”) and articulation disorders hampered intelligibility. However, above all, his speech was difficult to follow because of the difficulty to follow the sequences of the sentences or because of the lack of contextualisation, making it impossible to know whether A was relating to a real-life event or one he had seen on TV. For example, when asked to give five names of animals, his first answer was “hyenas” and then he continued with a rambling speech in which it was possible to perceive that he was referring to the Lion King movie. When asked again, he listed cow, lion, fox, and stork in reference to La Fontaine’s Fables, as if he had difficulty in detaching himself from his lived experience. When he was then asked to give the days of the week, he immediately replied “I do not know that animal” but could then, with the support of his mother, answer correctly. Similarly, when asked his age, he immediately replied “22 May,” but immediately snapped back to say “there is no May I know I know.” For more difficult questions (what does an orange have in common with a banana?), he promptly answered “sorry, my little friend” and then “sorry, madam,” and continued with a confused speech, but one could see that he was expressing that he could not answer in his own way: “I cannot shout.” During the interview, he showed twitching in his shoulders and when he spoke, his mouth could be deformed by a stretching of the left commissure.

### Case B

At about 2 to 3 years of age, B was always running with her arms slightly outstretched for balance, not stopping at any object or game, and obstinately emptying cupboards. Her eye contact was furtive but could land briefly on her parents. She would often bump into things and fall, but never cried. Pointing and some words appeared at about 3 to 4 years, but she did not appear to understand “no” or anger and laughed when she heard someone crying. Many everyday situations were ritualised (the front door always had to be opened by her mother). With the help of a communication program using signing, she became more receptive to gestures and started to use them to make requests, the appearance of “no” allowed her to express her refusal and thus reduce the behavioural problems. Sometimes she would repeat the last sound heard in echolalia.

Throughout her childhood, B required substantial support for learning, partially due to resistance and attentional difficulties. She had an aversion to changing locations: discussions were needed before each outing but once she had changed location, she seemed very happy. In terms of sensory perception, she showed tactile irritability: she appeared to be bothered by the wind touching her face and needed to touch her face as if to “counteract” this hypersensitivity. She was frequently prone to nausea and abdominal pains.

When B was around the age of six, she usually responded with short sentences and had difficulty in understanding instructions that included notions such as “under” or “beside.” B never spontaneously said “hello.” Each time her mother gently reminded her that she should say “hello,” B smiled from the corner of her mouth but could not say “hello.” At the age of 9 years, a medical report noted that “it was difficult to know what she was thinking, as her language difficulties combined with a repetitive laugh whenever she seemed to be in difficulty formed a façade behind which she hid. This laughter contrasts even more with the fact that her face rarely expresses her emotions, appearing blank. There are moments when she appears sad but she refuses to admit it. Her movements appear jerky, as if robotic.” She showed echopraxia and, sometimes, there were singularities in the attitudes that appeared unnatural (open right hand supporting the bottom of the face, as if to think), as well as brief episodes of rapid blinking of the eyelids. During her childhood, she liked to participate in many activities, such as horse riding, skiing, and drawing.

Around the age of 15, her abdominal pains became more intense and were accompanied by agitation, loss of appetite, and weight loss, leading to hospitalisation. Exhaustive investigations for infectious syndrome, inflammatory syndrome, malabsorption syndrome, chronic inflammatory bowel disease and porphyria were negative. Treatment with cyamemazine, risperidone, diazepam and zolpidem allowed a return to normalcy with the disappearance of pain and agitation, which lasted more than 6 months. At the same time, intermittent episodes of dystonic-like abnormal movements of the right leg associated with a relatively stereotyped dyskinetic component appeared, leading to several consultations with neurologists, who were unable to provide an explanation or diagnosis (Supplementary Video 3). These movements disappeared after 1 year.

At the age of 16, she presented with a state of agitation, with logorrhoea, wandering, and exaltation for several days. Then, the following month, she gradually became mute, except for a few phrases repeated in a loop, refused proposed activities, and showed a certain perplexity. As the weeks went by, clastic crises started to occur when she was solicited, along with thymic lability with unmotivated crying and then a stiff neck and a frozen smile, as well as urine and feces incontinence day and night. In view of the worsening of the catatonic signs (negativism, refusal to eat, weight loss, maintenance of posture, permanent tachycardia), she was hospitalised and treated with lorazepam (12 mg), which partially improved the symptoms, essentially negativism and communication, but a tendency towards restlessness and urinary incontinence remained.

B’s state alternated between short periods during which she had a frozen appearance, quasi-mutism, waxy flexibility, tremors, and echopraxia, and periods of major agitation with motor excitation, a loud quavering voice, dysphoria, sleep disturbed by incessant trips to the toilet, and abdominal pain. Her activities were very restricted: she picked up small dust balls and she filled the space on the pages by copying the same sentence. Although her facial expressions were difficult to decipher, she could show comforting gestures towards her loved ones. Her condition remained extremely variable. Sometimes the right half of her body appeared to be blocked or she had a rictus that sometimes “unblocked” suddenly with a scream. After several episodes of vagal faintness (extreme paleness that led her to lie down with her legs raised for 4 to 5 h), her condition has become permanently worse. She is currently mute, with posture maintenance (arms at 90°), and very tired, but responds to instructions by blinking her eyes. Most of the time, she stays in her chair without doing anything but can have impulsive acts or gestures (for example suddenly crosses a street, undresses unexpectedly, or disturbs everything in a room). She still manages to eat by herself and walk with her service dog, but needs help to wash herself.

### Case C

The first difficulties of C were identified at the age of 3½ when she entered kindergarten because of significant communication difficulties. C did not always answer questions or only answered in bits and pieces and it was difficult to know what she understood, but she always had a smile on her face. She observed other children but did not interact to them. She took little initiative and the teacher had to ask her to participate in most activities, except for singing and music, in which she eagerly joined.

At the age of six, she could not order the pictures in a story but could count to nine, read and write numbers to 10, name the days of the week, do puzzles, recite poetry, sing nursery rhymes, and write her name. C was passive in the classroom and continued her schooling until the age of 11. Her mother taught her to write by supporting her hand. She did faultless dictations. She knew the multiplication tables but could not use them to solve problems. She could read but read automatically without understanding what she was reading. She spoke in bits and pieces and did not always speak coherently but could make requests with her eyes. C could stay at home alone for 2 to 3 h, keeping herself busy with drawing.

From the age of 16, she stayed in a medicalised-educational boarding school. She had cyclical periods when she could appear calm or agitated. Agitated states were accompanied by screaming, body tensing, and sudden and rapid running away, which led to treatment with chlorpromazine, tiapride, and trihexyphenidyl. Her participation in activities fluctuated. In the cooking workshop, she could break eggs and use the electric mixer with verbal or even physical accompaniment, especially to start, but she also sometimes observed her friends throughout the group work without participating.

At the age of 20, she moved back home and the medication was gradually stopped. C lacks initiative and is therefore dependent on others for many acts of daily life. She cannot move around outside her home on her own. She eats by herself but needs help to cut her food and cannot prepare her meals. She knows how to dress herself but does not do so spontaneously and requires verbal instruction. She also needs reminders to go to the toilet. She can follow the instructions given but needs to be asked regularly. She is attentive to her surroundings but does not seek to interact or take the initiative to participate in what is happening around her.

In interviews, little spontaneous language is observed. C immediately smiles in response to the interlocutor’s smile. However, C responds inconsistently to verbal prompts. She looks into the eyes of others as if she wants to respond, looking alternatingly at her mother and the interviewer. We are not always sure if she has understood the question correctly. She often answers “no,” sometimes after a long latency period but without any oppositional behaviour. A few whispers are occasionally repeated several times. Sometimes she rubs her hands together for no apparent reason or “automatically” and repeatedly touches the desk.

From time to time, C starts to speak spontaneously for several minutes. Her speech is then expressive but continuous without pauses and is accompanied by mimics that are difficult to understand and follow (Supplementary data: transcription verbatim). These appear to occur mostly when someone else is present or when the TV is on. She also starts singing whenever she hears a song she knows (whether in French or English) on the radio or TV. Although she has very little initiative, she frequently engages in “tidying up” behaviour, which consists of either actually putting things in their place, (e.g., putting a pencil in the pencil cup) or moving objects around for no apparent reason, e.g., she will move an object from one end of the table to the other. She may also automatically pick up an empty glass and drink several times in a row until her mother takes it away.

There are periods of a few days or weeks when C is agitated and logorrheic, usually when she suffers from constipation. She no longer takes psychotropic medication and has never been hospitalised.

## Discussion

### Identify catatonic signs in childhood from a WKL school perspective

Psychomotor manifestations of three individuals with PMS were studied using methods from the WKL school. The findings show subtle and fluctuating qualitative abnormalities in psychomotricity affecting involuntary motor skills (reactive and expressive) that insidiously disrupted the behaviour, language, affectivity, and initiative of the affected individuals from childhood onwards, and which together constitute a ‘symptom-complex’ according to the WKL approach. The course evolved into a chronic form, with sub-acute exacerbations from adolescence onwards.

Such psychomotor manifestations may be minor and go undetected. Initially, in early childhood, they may appear insignificant (maintenance of a natural posture) relative to more disabling functional disorders (language delay for example). Their chronic nature (permanent smile, passivity) results in their being considered as part of the child’s temperament. Thus, such manifestations are not spontaneously reported by those around them and require direct observation by a clinician attentive to psychomotor manifestations to spot them. Then, from adolescence onwards, psychomotor signs may be neglected during phases of subacute manifestations and overshadowed by affective symptoms, prompting diagnoses of mood disorders. Finally, such manifestations may be difficult to perceive due to our fragmented knowledge of catatonia in terms of its definition and clinical description, which varies according to age, severity, and underlying mental disorder (77–79).

According to the DSM-5, the diagnosis of catatonia requires 3 of 12 signs that correspond to the signs most frequently observed among hospitalised catatonic adults (80). However, they do not fit well with the subtle clinical expression of our subjects in childhood. For example, our subjects may have an empty expressionless face rather than distorted facial expressions.

Catatonia can be expressed in various forms, agitated or stuporous, acute or chronic. The often-spectacular efficacy of benzodiazepines and electroconvulsive therapy (ECT) in acute stuporous catatonia, in contrast to its poor efficacy in patients with chronic catatonia, suggests both pathophysiological and psychopathological heterogeneity of this syndrome (81). Indeed, there are numerous diagnostic evaluation scales that differ in the choice of criteria used and the number of criteria necessary for a positive diagnosis of catatonia. Most focus on motor and behavioural manifestations, such as the Bush-Francis Catatonia Rating Scale (82), the Rogers Catatonia Scale (83), and the Bräunig Catatonia Rating Scale (84), whereas the scale of Northoff et al. (85) not only takes into account affective symptoms, in accordance with Kahlbaum’s conception, but also considers them to be intrinsic to catatonia and therefore required for the diagnosis.

All diagnostic assessment scales target more than 40 symptoms (motor, affective, behavioural, and vegetative) that may fluctuate over time and therefore require prolonged observation to be identified (79). Thus, catatonia is often underdiagnosed or confused with other diagnoses, even by experienced clinicians (86–90). Therefore, in our subjects, restlessness can be misdiagnosed as attention deficit with motor hyperactivity, mutism as a depressive symptom, and stereotypies as autistic traits if the context of these actions (intentional or not) is not considered.

The WKL framework allows detection of the subtle early psychomotor signs that lead us to reconsider the intentionality of other motor acts of these individuals, such as their behaviour, speech, or facial expressions. The semiological evaluation of motor signs is not always straight-forward and requires consideration of the contextual, qualitative, and developmental aspects of the symptoms in their analysis, as well as the use of hypothetical-deductive logic to interpret them. Negativistic behaviour can be the expression of ambitendence due to psychomotor impairment (psychomotor negativism) or indicate major thought inhibition, in which, in these cases, hypokinesia will dominate voluntary movements rather than expressive and reactive motricity, which is preserved (40).

The catatonic semiology described by the WKL school fits the clinical signs that are described in our three cases, such as proskinesia, parakinesia, pseudo-compulsion, “short circuit” responses, and ambitendence. The assessment and recognition of these signs requires familiarity and special knowledge that can be acquired through readings of Leonhard and educational courses. As Jack Foucher wrote (58): “The major, persistent obstacle to the diffusion of WKL phenotypes is their teaching …. But most problematic is the teaching of practical skills. Leonhard wrote about signs and symptoms that are unfamiliar if not completely unknown to the ICD/DSM world, hence remaining unnoticed or unexplored.”

Evaluation by the PCRS does not lead to a threshold-score for any of our three individuals. Indeed, this scale was constructed from a cohort of adolescents hospitalised either because of the severity of the catatonic syndrome or because of the severity of the associated co-morbidity (autoimmune or infectious encephalitis, etc.), which does not correspond to the profile of our subjects (76).

Our longitudinal study also reveals the developmental dynamics of catatonic semiology from childhood to adulthood in the context of *SHANK3* mutations. Thus, we can consider “automatically turning towards objects” as the childhood presentation of the equivalent utilisation behavior in adulthood. Moreover, the catatonic manifestations appear to be isolated in childhood, whereas they are more closely related to affective symptomatology in the subacute manifestations from adolescence onwards, suggesting the alternating and cyclical evolution described by Kahlbaum. Such a semiological modification from adolescence onwards could be linked to maturation of the prefrontal cortex and the alteration of the excitatory-inhibitory balance linked to the pruning of excitatory synapses due to the involvement of the SHANK3 protein in these synapses. This hypothesis would make it possible to reconcile, from a developmental perspective, the conceptions of diagnostic scales that privilege motor manifestations and those that also consider affective disorders and their underlying pathophysiological models: dysfunction of cortico-thalamo-cerebellar motor networks modulated by dopaminergic transmission for motor/behavioural catatonia rating scales versus dysfunction of higher-order frontoparietal networks insufficiently modulated by GABAergic and glutamatergic transmission for the Northoff catatonia rating scale (91).

The clinical pictures of these three individuals do not coincide with any of the simple forms of the WKL school of system catatonia. Rather, there is a mixture of signs (including psychomotor negativism, pseudo-compulsions, proskinesia, and speech-prompt responses) belonging to different forms of catatonia in each. It is possible that this corresponds to a combined form.

Furthermore, the presence of other features not belonging to system catatonia such as neurological or neurovegetative signs (hypotonia, delayed sphincter acquisition, sensory peculiarities, etc.) (92), leads us to consider that this is not an *isolated* form of chronic catatonia but a *syndromic* form.

Such “constitutional” dysfunction of the psychomotor centers disrupts education and interactions with the environment, which play a fundamental role in the child’s post-natal development of cognitive functions and learning due to the slowing of cerebral maturation, which is prolonged, particularly in the frontal regions, until 30 years of age. This may lead to severe repercussions in their learning, whether in terms of schooling, personal autonomy, or social communication skills, more as a result of educational difficulties than fixed biological impairment.

### Literature review on the association between catatonia and SHANK3 mutations

#### Prior studies supporting an association between catatonia and *SHANK3* mutations

##### Array-based comparative genomic hybridisation (array-CGH) analysis of cohorts of patients with catatonia reveals intragenic deletions of the SHANK3 gene

Two studies conducted a systematic array-CGH analysis of catatonic patients hospitalised in child psychiatry and adult psychiatry units and found one case of intragenic deletion of the *SHANK3* gene among 37 catatonic adolescents, i.e., almost 3%, and two cases among 15 catatonic adults with intellectual disability, i.e., 13% (68, 93). These significant results should encourage exome or gene panels sequencing that includes the *SHANK3* gene of patients from these cohorts with a normal CGH array to find *SHANK3* point mutations.

##### Case reports of patients with severe atypical behavioural disorders and catatonic manifestations whose organic work-up revealed SHANK3 mutations

Serret et al. (94) reported the case of two adolescents with a very similar clinical history. Both were diagnosed in childhood with ASD and presented with major behavioural changes during changes in residential care settings at around 12 to 13 years of age, with the loss of motor skills, language regression, and the loss of autonomy, suggesting a diagnosis of catatonia-like symptoms, as the Bush Francis Catatonia Rating Scale (BFCRS) was negative in one case. The work-up revealed a *SHANK3* point mutation in both patients.

Similarly, Bey et al. (16) reported the case of four adolescents hospitalised and treated by four independent teams for very similar subacute clinical pictures occurring at puberty, with mutism, hallucinations, insomnia, inconsolable crying, obsessive–compulsive behaviour, the loss of self-care, and urinary retention and/or incontinence. Three of them had a catatonic syndrome. After receiving various psychotropic drugs (antipsychotics, antidepressants) with little effect, followed by lorazepam, lithium and/or hormonal contraceptives, with variable responses, their condition improved with immunosuppressive treatment in the face of suspected autoimmune encephalitis, before whole-exome sequencing revealed that they carried a point mutation in the *SHANK3* gene. The patient without catatonic syndrome had “choreiform hand movements and tremor” and “near complete aphasia,” which were considered to be neurological signs but may represent unrecognised catatonic signs.

These observations highlight the interest of genetic investigations for individuals with a history of developmental delay and atypical behavioural disorders with catatonic manifestations (95, 96).

##### Psychiatric evaluation of cohorts of PMS individuals with disruptive behavioural disorders reveals catatonic manifestations

Verhoeven et al. (15) studied 24 PMS adults referred for diagnostic and therapeutic advice for recurrent manifestations of challenging behaviours coinciding with symptoms from the schizoaffective spectrum. The evaluation of these subjects by standardised neuropsychiatric examination revealed catatonic manifestations in 5 of 24 cases (20%).

Kohlenberg et al. (17) recruited 38 individuals through the PMS Foundation with psychiatric changes, such as mood episodes, psychosis, marked changes in sleep and energy, and significant loss of skills. Psychiatric disorders were assessed by a semi-standardised interview with the caregiver. Catatonic manifestations were found in 20 of 38 cases (53%).

Only a quarter of the participants were known to have PMS before the onset of psychiatric symptoms. The onset of psychiatric symptoms and/or regression prompted investigations, leading to a diagnosis of PMS, as in the cases reported in the previous paragraph. None of the individuals were under 13 years of age.

Thus, the results of these studies, which went from phenotype to genotype, converge with ours, which went from genotype to phenotype, reinforcing the validity of the association between catatonia and *SHANK3* mutations.

#### Prior studies may have missed the association between catatonia and *SHANK3* mutations

Other publications on *SHANK3* mutations do not report catatonic manifestations. There are several explanations for these negative findings, which we discuss below while highlighting the difficulty of diagnosing catatonia, whether in cohort studies using standardised tools that do not target catatonia, or in in-depth case studies due to gaps in our knowledge of chronic, childhood and mild forms.

##### Catatonia mistaken for atypical bipolar disorder due to a failure to detect muscular signs (measurement and observation bias)

The two previous studies of Verhoeven et al. (15) and Kohlenberg et al. (17) did not find catatonic manifestations in most of the participants: 80% of the cohort of Verhoeven et al. (15) and a little less than 50% of that of Kohlenberg et al. (17). However, they both underlined the importance of atypical mood disorders (“atypical bipolar disorder,” “mood lability,” “mania and then depression”) starting after adolescence in more than 75% of the participants, confirmed by numerous case reports (96–101).

It is possible that the observed atypical bipolar disorder corresponds to the alternating and cyclical evolution of catatonia described by Kahlbaum, i.e., with successive phases of melancholy, mania, stupor, and then confusion, and why the muscular signs would have gone unnoticed or not taken into account. Indeed, five of the 18 subjects in the cohort of Kohlenberg et al. (17) considered to be *without catatonic manifestations* had motor manifestations of “posturing” (case 3), “hand posturing and gait disturbance” (case 4), and “choreiform movements” (case 13), as well as pica behaviours (cases 33, 34), which are difficult to interpret in the absence of context but which could correspond to an abnormal tendency to initiate movements automatically in response to external stimulation (proskinesia). The lack of consideration of motor manifestations may have been due to the assessment method, which was a standardised interview with the caregiver, who is likely to complain more about symptoms such as agitation, impulsive aggression or bolting, and eating non-food items than non-disabling signs (empty expressionless face). This may also have been due to gaps in the recognition of catatonic signs among clinicians. Direct observation and clinical examination of the individual by trained clinicians are essential in the search for muscular signs (90, 102).

##### Failure to diagnose catatonia due to a lack of consideration of the circumstances

Several clinical case reports or cohorts of children with PMS have been reported, most diagnosing ASD (11, 103–106) or a subtype of ASD, such as Rett syndrome-like (10) or Heller’s disintegrative disorder (12), but none mentioned catatonic phenomena.

In 2015, we published the clinical case of an 8 year-old girl with Heller’s disintegrative disorder carrying an intragenic deletion of the *SHANK3* gene without mentioning catatonic manifestations, although we were already aware of the evolution towards catatonia in adolescence in PMS (12). As in the other publications (10, 11, 103–106), we detailed this little girl’s incessant restlessness: ‘difficulty focusing her attention’, ‘increasing restlessness’, and ‘rapid shifts from one unfinished activity to another’ but without describing the circumstances of the restlessness.

The frequency of motor hyperactivity in neurodevelopmental disorders, the polythetic diagnostic approach based on an atheoretical descriptive semiology, the disposition of the human mind to consider actions as the expression of individual intentions, the lack of knowledge of chronic forms of childhood catatonia, and, above all, the absence of consideration of the context of the symptoms (Falret’s third principle (36)) explain why we failed to consider this incessant restlessness as an unintentional action that could have made us consider it as a qualitative impairment of psychomotricity. The context of this girl’s activities was always aimless. It is clear to us now, in retrospect, that this was consistent with automatic object rotation syndrome, which characterises proskinesia in childhood.

Taking into account the contextual, qualitative, and developmental aspects of symptoms generally allows an understanding of their nature and the ability to differentiate between related symptoms, in particular by questioning their intentionality in the motor, speech, or behavioural domains. As Nordgaard et al. (70) remind us, «we always perceive expression (sign) in the context of its temporal unfolding and in conjunction with the expressed contents (symptoms) and vice versa … [there is] the crucial difference between perceiving what may be the very same physical movement as a wink versus as a mere blink, depending on context and ascribed expression or intent.”

Nevertheless, this allowed us to postulate that Heller’s disintegrative disorder, which we diagnosed, is a form of chronic catatonia, as has already been suggested (107, 108).

##### Failure to diagnose catatonia due to measurement bias

Nevado et al., (32) studied the clinical phenotype of a cohort of 210 PMS individuals using items from the Human Ontology Phenotype (HPO) database which contains 13,000 terms but not “catatonia,” whereas pseudo-dystonic parakinesia (“snout spasm”) can be evoked in the photo of the first patient of this study.

The behavioural phenotype of three other cohorts, an American cohort of 170 individuals (109), a Spanish cohort of 60 individuals (110), and a Dutch cohort of 33 children (111), was studied using standardised tools that assess autistic symptomatology (ADI-R, ADOS-2), cognitive level, language level, adaptive functioning (Vineland), and behavioural disorders, such as the Child Behavior Checklist (CBCL), etc. The data were studied using descriptive and inferential statistical analyses. None of the tools used mention psychomotor or catatonic signs.

In these studies, the origin of not having seen the psychomotor phenomena (which makes it possible to see the “snout spasm”) is not so much an observational bias but rather a measurement bias, insofar as the tools used were not adequate to evaluate psychomotor manifestations.

##### Selection bias may explain that mild forms of catatonia are missed

Samogy-Costa et al. (112) published the case of a 20 year-old male described as being atypical of the PMS profile due to having a normal IQ and being less functionally impaired than typical PMS patients. Functional impairments included failure of the psycho-technical test for the driving license, attentional problems, difficulties related to making and keeping friends, social and emotional responsiveness, processing figured speech, sensory sensitivity, and the insistence on sticking to routine.

The authors did not specify the nosological framework of these manifestations nor mention catatonia. In the absence of further observations and with an understanding approach based on the narrative of the patient and his relatives (113), one can only speculate on the underlying mechanisms. Difficulties in communication and socialization can be related to a lack of interest in relationships (schizoid personality), difficulties in emotional expression (alexithymia, autism, catatonia), a lack of social experience (socio-economic difficulties, educational deficiency, associated somatic conditions, etc.), a disorder of executive functions, a fear of the judgement of others (social phobia), qualitative abnormalities of psychomotricity that affect expressive and reactive involuntary motricity (catatonia). Thus, in the latter case, latency in answering questions could explain the failure in timed driving tests and the lack of initiative in conversation could have made it difficult to maintain relationships and a certain degree of opposition could have made them confrontational.

Consistent with the conceptual framework proposed in this work and in accordance with the principle of parsimony, we propose that the patient had a mild form of catatonia. Further observations of this subject’s behaviour and his subjective experience would help to support this hypothesis.

The rarity of such cases as this may be due to a selection bias, as genetic investigations (CGH-array, exome or genome sequencing) are still rarely offered as part of the diagnostic routine to people with a neurodevelopmental disorder and a normal IQ.

### Strong comorbidity between features of autism features and catatonia or a singular disorder more fundamentally linked to SHANK3 mutations?

A review of literature shows that catatonic signs are identified in more than half of subjects with a SHANK3 anomaly after adolescence (17), whereas they are virtually never identified as such in childhood, either are not sought (32), are not known (112) or, more interestingly, are misinterpreted (12).

Furthermore, ASD is very common (>70%) among individuals with SHANK3 abnormalities. Qualitative analysis of the ADI-R and ADOS scores showed differences from those obtained in autism. For example, our three individuals also had positive cut-off scores for the social interaction and communication domains of the ADI-R but not for repetitive and restricted behaviour (RRB) domain. In addition, individual 1 had a score of zero for the RRB domain of the ADOS-2 (Table 1), even though no minimum threshold is required for this domain since it is the addition of the score for the “social affect” domain and the score for the RRB domain that is taken into account by the algorithm to compare this total score with the thresholds for autism or ASD. Similarly, Ponson et al. analysed the RRB of PMS subjects in detail by separating sensorimotor RRBs corresponding to unusual sensory interests and repetitive physical mannerisms, likely to improve over time and strongly associated with cognitive abilities, and RRBs characterized by resistance to change also known as “insistence on sameness,” corresponding to compulsive behaviours (negative reactions to changes in the environment or an unusual attachment to objects) (20). However, ‘insistence on sameness’ items do not appear in the diagnostic algorithm of the ADI-R. Using a RRB scale, Ponson et al. showed that the “sameness” dimension was globally missing among PMS patients and therefore, they do not meet the current DSM5 criteria for a diagnosis of ASD. This led them to discuss the diagnosis of social communication disorder, even if it does not take catatonic symptoms into account (20).

Finally, the frequency of catatonic development in adolescence is very high among subjects with a SHANK3 anomaly, versus only 10% among subjects diagnosed as autistic in childhood (114).

Therefore, there is high comorbidity between catatonic symptoms and autistic traits, meaning these disorders always go hand in hand. According to the ‘reverse phenotyping’ approach based on the principle of causality, it seems appropriate to consider them as belonging to a *single* disorder with a common physiopathology and psychopathology, despite their highly variable clinical presentations (the aimless agitation of A appears to contrast with the lack of initiative of C in childhood). Thus, this very rare and novel phenotype related to mutations in the SHANK3 gene is close to the “early-onset catatonia” described by the WKL school.

### Early-onset catatonia: the importance of considering qualitative psychomotor abnormalities in the differential diagnosis of Kanner’s autism

Shorter and Wachtel (115) found that “Kanner’s young patients were drenched in catatonic symptoms” and searched for the presence of catatonic symptoms in the 11 children described by Kanner in his seminal article (8). Curiously, they did not find them in the case of Donald (case 1), which was by far the most detailed case and considered to be the prototypical case of autism (116), but did find them in several other cases (cases 3, 4, 5, 7, 8, 10), especially that of Elaine (case 11), which we describe in detail below. Taking the context into account, as Kanner explains, allows us to distinguish very different phenomena between Donald and Elaine for identical symptoms, such as the stereotyped repetition of words.

Thus, Donald uttered words or phrases such as “chrysanthemum,” “dahlia, dahlia, dahlia,” “business,” “trumpet vine,” “The right one is on, the left one is off,” “Through the dark clouds shining.” Elaine had seemingly similar utterances: “Dinosaurs do not cry,” “crayfish, sharks, fish, and rocks”; “Crayfish and forks live in children’s tummies”; “Butterflies live in children’s stomachs, and in their panties, too”; “Fish have sharp teeth and bite little children.” Kanner specified that Donald “seemed to have much pleasure in ejaculating words or phrases,” whereas Elaine’s speech “is never accompanied by facial expression or gestures,” highlighting a qualitative anomaly in Elaine’s expressive psychomotricity (117).

Furthermore, at nursery school, Elaine “drank the water and ate the plant when they were being taught to handle flowers,” corresponding to a pica that can be related to an environment-dependent behaviour of proskinesis. This type of proskinetic behaviour was not reported in Donald.

In 1971, Kanner reported on the fate of these 11 children, and it turned out that Donald’s evolution was quite different from that of Elaine (118). At the age of 36, Donald lived with his parents. After graduating from college, he had worked as a bank teller for 12 years. His principal pastime was golf, which he played 4 to 5 times a week at the local club. He was even-tempered but knew what he wanted. He liked his independence and participated little in social conversations. He had never taken any medication.

Elaine, aged 31, had lived in a psychiatric hospital since she was 11 years of age. She was able to take care of her personal needs. Her speech was slow and occasionally unintelligible and she was manneristic. Her social contact skills were poor. She could not participate in a conversation, except to express her immediate needs. If things did not go her way, she became acutely disturbed, yelling, hitting her chest with her fist and her head against the wall. In her lucid periods, however, she was cooperative, pleasant, childish, and affectionate (118).

Leonhard was well aware that his description of the children applied to some of the autistic children described by Kanner (119), especially Elaine as a case-in-point. In re-reading Elaine’s case, it appears to us, that, unlike Donald, she had alterations in involuntary processes and an unfavourable evolution more suggestive of early-onset catatonia.

### Limitations of the study

This exploratory case study aimed to highlight catatonic manifestations from childhood and is preliminary, requiring further specific identification of chronic catatonia in children.

Our study had several biases. First, there was a selection bias, as it concerned only three participants and it is therefore necessary to confirm our clinical observations on a larger number of subjects. In addition, there was measurement bias, as part of our observations were made retrospectively. Prospective studies are therefore warranted.

## Clinical and research implications

It is well documented that there is symptomatic overlap between the clinical features of autism and catatonia, which can lead to diagnostic confusion (120), especially as an evolution towards catatonia is reported in adolescence for 10% of subjects diagnosed with autism in childhood (55, 114, 121). Burns et al. (122) reported an intriguing case of a subject diagnosed with autism in childhood in which the onset of excited catatonia led to the prescription of ECT, which not only treated the episode of acute catatonia but also motor symptoms present since early childhood that were considered to be autistic self-stimulation, highlighting the difficulty of attributing well-identified behaviours to psychopathological conditions.

This subgroup is probably clinically and aetiologically heterogeneous, and there are several possible hypotheses to explain this course, each explaining a fraction of the subjects in this subgroup. It is possible that autism and catatonia are two clinical expressions of the same psychopathological entity (123, 124) or there is a subtype of autism that could be called “autistic catatonia” (122, 125, 126). Furthermore, neurodevelopmental disorders could be a risk factor for developing catatonia (127) or they could correspond to early-onset catatonia, the signs of which are not perceived in childhood.

Our study provides some support for this last possibility and encourages to read Leonhard’s “Early childhood catatonia” to identify these subtle and fluctuating catatonic signs in childhood (57).

The assessment of psychomotor abnormalities remains a challenge in clinical practice. The main obstacle lies in distinguishing motivational symptoms from automatic actions, which can be complex in such children whose language development is often poor. In addition, the assessment of muscle tone (gegenhalten or mitgehen) may also be difficult due to comprehension problems.

In-depth analysis of filmed interviews of these subjects could help to finely identify subthreshold and transient catatonic symptoms, taking into account the psychological context of the individual. For example, the analysis of the conditions of occurrence of immediate echolalia would allow a better understanding of its different meanings, and to consider in some cases, that it could be a utilisation behaviour similar to proskinesia (128, 129). In addition, use of video could facilitate the evaluation of parakinesias by allowing an *a posteriori* re-examination of psychomotricity by cutting the sound (64, 65). We believe that this approach is necessary to disentangle the symptomatic overlap between catatonia and autism.

As the diagnosis of early-onset catatonia is still highly challenging, clinical studies are needed to continue Leonhard’s work to better identify the catatonic symptoms and to develop diagnostic tools similar to the ADI-R for autism to detect early-onset catatonia.

SHANK3 mutations are rare, but the prospective study of children who have benefited from early genetic diagnoses could make it possible to identify early subclinical signs of catatonia that would be useful for detecting other forms of early-onset catatonia (130).

Furthermore, the worsening of catatonic symptomatology from adolescence onwards makes it necessary to carry out studies with at least several years’ hindsight to evaluate different therapeutic strategies (lithium, neuromodulation, immunosuppression), given the cyclical nature of these manifestations, which can lead to the clinical improvement following therapeutic intervention being confused with the natural history of the neurodevelopmental disorder (100).

## Conclusion

New-generation sequencing techniques are identifying an increasing number of mutations in the genes responsible for neurodevelopmental disorders, providing a better understanding of their pathophysiological basis, but providing little insight into their psychopathology.

The possibility of validly grouping subjects who share the same rare mutation and studying their psychopathological phenotype opens a new field of research for the psychopathological characterisation of these genomic anomalies, even if this grouping is generally not sufficient to understand their psychobehavioural manifestations. The difficulty in establishing correlations between psychobehavioural symptoms and genomic anomalies illustrates the particularity of psychiatric semiology relative to somatic semiology, as the relationship between semiology and psychopathology is less linear than between the semiology and physiology of somatic diseases (131, 132).

The identification of a rare pathogenic variant shared by several individuals forces us to search for a conceptual framework to provide semiological coherence within their psychobehavioural manifestations, enabling us to consider that they constitute a single nosological entity and to put forward the hypothesis of a homogenous physiopathology and psychopathology.

Using the fine semiology developed by the WKL school concerning systemic catatonias, this approach has enabled us to show that individuals carrying an intragenic mutation or deletion of the SHANK3 gene present qualitative psychomotricity anomalies that affect involuntary motor skills (reactive and expressive), which insidiously disrupt the behaviour, language, affectivity, and initiative of affected individuals from childhood onwards, progressing to a chronic form with sub-acute exacerbations from adolescence onwards.

The conception of the WKL school of catatonia as its consisting of qualitative alterations in involuntary motor actions that affect expressive and reactive motricity that have the character of intentional acts but are performed without the influence of the will, allows for the perception of a homogeneous psychopathology among these individuals. This is true even if they show clinical variability resulting from a complex interplay between their own genetic background, environmental influences, and coping strategies, which can be expressed in many ways, such as seemingly illogical responses for A (short-circuit responses), an inability to say hello for B (ambitendence), and utilisation behaviour for C (proskinesia).

Our results provide a new perspective on these individuals, calling for caution in the interpretation of their presenting symptoms. For example, a sad face does not necessarily mean sadness, rambling speech does not automatically have to evoke a thought disorder, and failure on psychometric tests does not inevitably indicate impairment.

The pathophysiological link between altered SHANK3 protein function and catatonic manifestations is yet to be discovered. The development of neuroimaging techniques that can instantly capture both the neuronal activity and metabolic activity of nervous tissue could make it possible to decipher the dynamic dysfunction of neuronal networks (133, 134).

Finally, a better understanding of the psychopathological phenotype associated with mutations in the *SHANK3* gene can help in the clinical interpretation of unknown missense and splice variants identified by NGS.

Initially focused on a rare genetic disease, our work contributes to improving the diagnostic evaluation of more common developmental disorders, such as autism spectrum disorders, by showing the interest of considering qualitative abnormalities of psychomotricity that affect expressive and reactive involuntary motricity, regardless of the ADI and/or ADOS scores, often neglected relative to cognitive, emotional, and socio-communicative symptoms, to differentiate early-onset catatonia from autism.

## Data availability statement

The original contributions presented in the study are included in the article/Supplementary material, further inquiries can be directed to the corresponding author.

## Ethics statement

Ethical review and approval was not required for the study on human participants in accordance with the local legislation and institutional requirements. Written informed consent has been obtained from the individual(s), and minor(s)’ legal guardian/next of kin, for the publication of any potentially identifiable images or data included in this article.

## Author contributions

AP designed the study and wrote the first version of the manuscript. DD, CB, CL-L, CR, and LR added their modifications, assisted with manuscript development, and participated in revising the manuscript. MN analyzed of the audio recordings and completed the transcriptions. All authors contributed to the article and approved the submitted version.

## Conflict of interest

The authors declare that the research was conducted in the absence of any commercial or financial relationships that could be construed as a potential conflict of interest.

## Publisher’s note

All claims expressed in this article are solely those of the authors and do not necessarily represent those of their affiliated organizations, or those of the publisher, the editors and the reviewers. Any product that may be evaluated in this article, or claim that may be made by its manufacturer, is not guaranteed or endorsed by the publisher.

## Footnotes

^1^*G*egenhalten is an oppositional paratonia in which the patient unconsciously resists movements imposed on him/her (e.g., resists bending or passive extension of the arm).

^2^*M*itgehen is a condition in which the patient can no longer oppose an instruction or order, whether verbal (automatic obedience) or motor (patient unable to oppose a movement despite being instructed to resist).
