# Supplementary material for: Early-onset catatonia associated with SHANK3 mutations: looking at the autism spectrum through the prism of psychomotor phenomena
Source: Front Psychiatry. 2023 Sep 22;14:1186555. doi: 10.3389/fpsyt.2023.1186555 (PMC10557257; doi:10.3389/fpsyt.2023.1186555)
Supplement: Supplementary file 1 [file Data_Sheet_1.PDF]

no I don't feel like it /
well, we'll see /
xxx can't go [/] can't go because it's not time, right?
but it's time, what are we going to do xxxx at noon /
I'm not going now /
I'm going to Arco because I'm not going to see my Kareen so I don't get the first one /
I don't feel like it /
well, I'm wondering /
you talk about what you did /
you didn't /
you did /
uh to see France /
what did you do / what did he do /
he did on Kareen he didn't finish /
um there you go /
I can't now /
yes he tells me [/]that I would have the impression in fact quite normal /
for you I called during the twentieth /
the twentieth i(l) won't be there /
i(l) will be /
so I don't have /
of xxx [/] of Kareen /
she doesn't want to go all the way to France /
she doesn't want to go to Kareen /
from earlier she won't go /
she won't go /
I'll take her here /
she doesn't want to go to Kareen /
not France I'm over 20 I can't / no me no Kareen /
we won't see your feet /now we'll see you /
we won't talk to you /
we'll tell you /
no more pressure already /
no night /
no one but me /
so I don't have /
and I see you /
that you what we see how you are like that /
not a franc of Kareen’s cleo /
see xxx /
already you've waited almost 20 years /
I can't /
I would have xxx and all that /
but you I didn't love [/]
/but no but no/
